# An Integrative Review of the Cardiovascular Disease Spectrum: Integrating Multi-Omics and Artificial Intelligence for Precision Cardiology

**DOI:** 10.3390/diseases14010031

**Published:** 2026-01-13

**Authors:** Gabriela-Florentina Țapoș, Ioan-Alexandru Cîmpeanu, Iasmina-Alexandra Predescu, Sergio Liga, Andra Tiberia Păcurar, Daliborca Vlad, Casiana Boru, Silvia Luca, Simina Crișan, Cristina Văcărescu, Constantin Tudor Luca

**Affiliations:** 1 Doctoral School, “Victor Babes” University of Medicine and Pharmacy, Eftimie Murgu Square 2, 300041 Timisoara, Romania; gabriela.tapos@umft.ro; 2 Arad County Clinical Emergency Hospital, 310037 Arad, Romania; 3 “Victor Babes” University of Medicine and Pharmacy, 2nd Eftimie Murgu Square, 300041 Timisoara, Romania; iasmina-alexandra.predescu@umft.ro (I.-A.P.); sergio.liga96@gmail.com (S.L.); andra.pacurar@umft.ro (A.T.P.); 4 Research Centre for Pharmaco-Toxicological Evaluation, Faculty of Pharmacy, “Victor Babes” University of Medicine and Pharmacy, 2nd Eftimie Murgu Square, 300041 Timisoara, Romania; 5 Department of Applied Chemistry and Engineering of Organic and Natural Compounds, Faculty of Chemical Engineering, Biotechnologies and Environmental Protection, Politehnica University Timisoara, Vasile Pârvan No. 6, 300223 Timisoara, Romania; 6 Department of Biochemistry and Pharmacology, Faculty of Medicine, “Victor Babes” University of Medicine and Pharmacy, 2nd Eftimie Murgu Square, 300041 Timisoara, Romania; vlad.daliborca@umft.ro; 7 Faculty of Medicine, “Vasile Goldis” Western University of Arad, 86 Liviu Rebreanu Street, 310048 Arad, Romania; 8 Cardiology Department, “Victor Babes” University of Medicine and Pharmacy, 300041 Timisoara, Romania; silvia.luca@umft.ro (S.L.); simina.crisan@umft.ro (S.C.); cristina.vacarescu@umft.ro (C.V.); constantin.luca@umft.ro (C.T.L.); 9 Institute of Cardiovascular Diseases Timisoara, 300310 Timisoara, Romania; 10 Research Center of the Institute of Cardiovascular Diseases Timisoara, 300310 Timisoara, Romania

**Keywords:** cardiovascular disease spectrum, heart, integrative review, precision cardiology

## Abstract

Background/Objectives: Cardiovascular diseases (CVDs) remain the leading cause of morbidity and mortality worldwide and increasingly are recognized as a continuum of interconnected conditions rather than isolated entities. Methods: A structured narrative literature search was performed in PubMed, Scopus, and Google Scholar for publications from 2015 to 2025 using combinations of different keywords: “cardiovascular disease spectrum”, “multi-omics”, “precision cardiology”, “machine learning”, and “artificial intelligence in cardiology”. Results: Evidence was synthesized across seven major clusters of cardiovascular conditions, and across these domains, common biological pathways were mapped onto heterogeneous clinical phenotypes, and we summarize how multi-omics integration, AI-enabled imaging and digital tools contribute to improved risk prediction and more informed clinical decision-making within this spectrum. Conclusions: Interpreting cardiovascular conditions as components of a shared disease spectrum clarifies cross-disease interactions and supports a shift from organ- and syndrome-based classifications toward mechanism- and data-driven precision cardiology. The convergence of multi-omics, and AI offers substantial opportunities for earlier detection, individualized prevention, and tailored therapy, but requires careful attention to data quality, equity, interpretability, and practical implementation in routine care.

## 1. Introduction

Cardiovascular diseases (CVDs) remain the leading cause of mortality and morbidity worldwide, accounting for nearly one-third of global deaths and imposing a profound socioeconomic burden on healthcare systems [1,2]. Despite significant progress in pharmacological therapy, interventional cardiology, and preventive strategies, major unmet needs persist in early detection, individualized risk prediction, and long-term disease prevention [3,4].

Recent progress in genomics, bioinformatics, and digital technologies has reshaped our understanding of cardiovascular pathophysiology, revealing complex interactions between molecular, cellular, and systemic processes [5,6]. While traditional clinical classifications treat cardiovascular conditions as distinct entities, extensive evidence shows that they share interconnected biological mechanisms [5,6,7,8]. These interrelationships form the foundation of the cardiovascular disease spectrum, a unifying framework in which diverse cardiovascular disorders coexist, influence one another, or evolve sequentially across the lifespan. Shared mechanisms such as endothelial dysfunction, chronic inflammation, oxidative stress, vascular remodeling, thrombotic imbalance, and genetic susceptibility further reinforce this integrative perspective [7,8,9,10,11].

**Figure 1:**
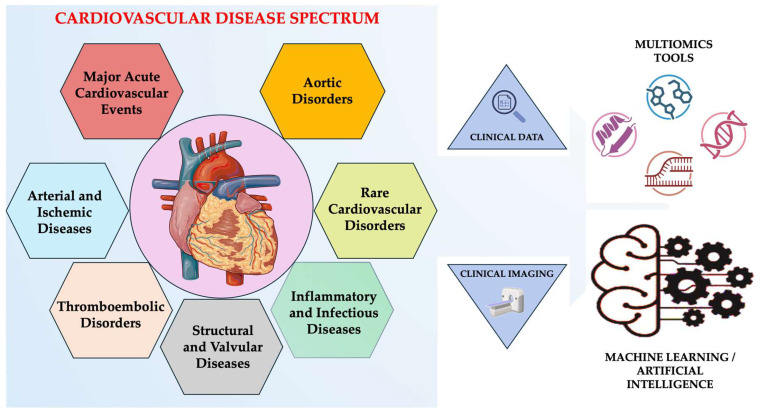
Integrated view of the cardiovascular disease spectrum. Created in BioRender. Țapoș et al. (2026) https://www.biorender.com/ (accessed on 11 November 2025).

In parallel, large-scale genome-wide association studies (GWAS), next-generation sequencing (NGS), and polygenic risk models have revealed both rare monogenic mutations and common polygenic architectures underlying coronary artery disease, cardiomyopathies, arrhythmias, and vascular disorders [7,8]. Similarly, multi-omics approaches (e.g., genomics, transcriptomics, proteomics, metabolomics, epigenomics) have given a system-level view of cardiac biology, identifying novel biomarkers and therapeutic targets that go beyond traditional diagnostic boundaries [9,10,11]. Artificial intelligence (AI) and machine learning (ML) have become crucial tools in cardiology, able to process high-dimensional datasets from imaging, electrocardiography, and clinical records in parallel with these molecular advances [12,13]. AI algorithms are now enabling automated image interpretation, predictive modeling, and phenotype clustering with unprecedented accuracy, which aids in early diagnosis and personalized intervention [13,14]. These technologies facilitate automated image interpretation, phenotype clustering, early disease detection, and predictive modeling, enabling earlier and more personalized interventions (Figure 1) [15,16,17].

The convergence of precision medicine, multi-omics, and AI is redefining cardiovascular medicine by bridging molecular discoveries with clinical decision-making and supporting a shift toward predictive, preventive, and personalized care [18,19,20]. However, implementation remains challenged by data heterogeneity, ethical concerns, and regulatory complexity [21,22]. Understanding these interdisciplinary connections is crucial for converting scientific innovation into tangible patient benefits. Also, a clinically useful spectrum-based framework therefore requires not only mechanistic integration, but also pragmatic guidance on validation, interpretability, and workflow adoption.

The review presents current research on major cardio-vascular diseases, emphasizing shared mechanisms, cross-disease interactions, and the growing importance of advanced technologies in reshaping cardiovascular diagnostics and therapies. The rationale for examining a broad range of cardiovascular conditions within a single conceptual narrative is clearly explained in this integrated framework, while also reflecting the complexity of modern cardiovascular medicine. We further distinguish approaches that are currently ready for routine clinical deployment from those that remain emerging or investigational, and we outline key requirements for hospital implementation, including standardization and interpretability. Finally, we propose a pragmatic roadmap for research and adoption, emphasizing that AI and multi-omics are intended to augment, but not replace the clinical history-taking, examination, and clinical judgment.

## 2. Sources and Search Strategy

The search of scientific literature was performed in PubMed, Scopus, and Google Scholar covering scientific publications from 2015 to 2025. Keywords included: “cardiovascular disease spectrum”, “multi-omics”, “precision cardiology”, “machine learning”, and “artificial intelligence in cardiology”. The use of reference list screening was extended to identify complementary studies that were relevant to clinical integration across the cardiovascular disease spectrum. In addition, previous landmark studies published before 2015 were included when they were clinically pivotal. To operationalize the cardiovascular disease spectrum for narrative synthesis, we organized the included evidence into seven clinical–mechanistic clusters selected to reflect frequent clinical overlap. The review is structured into: (i) atherosclerotic and ischemic cardiovascular diseases; (ii) thromboembolic disorders; (iii) structural and valvular diseases; (iv) inflammatory and infectious cardiovascular diseases; (v) aortic disorders (aortic aneurysm and dissection); (vi) rare cardiovascular disorders; and (vii) major acute cardiovascular events. This clustering is intended as a pragmatic organizing framework rather than a new taxonomy, and cluster boundaries are permeable to reflect real-world multimorbidity.

## 3. Cardiovascular Diseases

### 3.1. Arterial and Ischemic Diseases

#### 3.1.1. Atherosclerosis and Arteriosclerosis

Atherosclerosis and arteriosclerosis are often used interchangeably, but they represent distinct vascular conditions with overlapping features. Arteriosclerosis is a broad term describing the hardening and loss of elasticity of arteries, typically associated with aging. In contrast, atherosclerosis is a specific type of arteriosclerosis, characterized by the build-up of lipid-laden plaques in the intimal layer of medium and large arteries, driven largely by inflammation and metabolic dysfunction [23,24].

Atherosclerosis is a chronic, progressive inflammatory disease marked by endothelial dysfunction, lipid accumulation, and immune cell infiltration in the arterial wall. It begins with endothelial injury, often triggered by hypertension, smoking, hyperlipidemia, or disturbed blood flow, followed by the accumulation of modified lipoproteins (especially oxidized LDL), monocyte adhesion, and transformation into foam cells, leading to fatty streaks [25,26,27,28]. In contrast, arteriosclerosis typically results from age-related degeneration of the arterial media, involving elastin fragmentation, collagen accumulation, and calcification, particularly in large elastic arteries like the aorta. It leads to arterial stiffening rather than luminal occlusion and is reflected in increased pulse wave velocity (PWV) [23,24]. At the mechanistic level, inflammation represents a central process underlying atherosclerosis progression. Inflammation is the body’s response to injury or infection and is mediated by components of innate immunity, such as neutrophils and macrophages. These signals are recognized by pattern recognition receptors (PRRs), which identify PAMPs and DAMPs and initiate inflammatory responses. PRRs, including TLRs, NLRs, RLRs, and CLRs, are expressed in innate immunity cells and have distinct localizations and specificities. Among these, NLRP3 recognizes DAMPs and forms the NLRP3 inflammasome in the cytosol. Among pattern recognition receptors, the NLRP3 inflammasome has attracted particular interest due to its central role in inflammatory responses [29]. The NLRP3 inflammasome plays an essential role in immune defense against bacterial, fungal, and viral infections, but its dysregulation is involved in the pathogenesis of several inflammatory diseases, including atherosclerosis, thus contributing to its progression. Structurally, NLRP3 is a tripartite protein consisting of the pyrin domain (PYD), the central nucleotide-binding and oligomerization domain (*NOD*/*NACHT*), and the C-terminal domain rich in leucine repeats (LRR) [30]. In this pathological context, the NLRP3 inflammasome plays an important role in the pathogenesis of atherosclerosis, being involved in endothelial cell damage and in the link between lipid metabolism and the inflammatory response. Activation of the NLRP3 inflammasome causes the secretion of IL-1β and IL-18, which amplifies inflammation and oxidative stress, contributing to endothelial dysfunction. In atherosclerotic plaques, NLRP3 is predominantly expressed in macrophages and foam cells, where it is activated by ox-LDL and cholesterol crystals, promoting inflammatory responses and macrophage differentiation towards the M1 phenotype. Foam cells, derived from macrophages and vascular smooth muscle cells, release DAMPs in advanced lesions, which maintains NLRP3 inflammasome activation and contributes to atherosclerosis progression [31]. Beyond atherosclerosis, activation of the NLRP3 inflammasome has been shown to contribute to myocardial ischemia–reperfusion injury and chronic heart failure. In this context, NLRP3-dependent caspase-1 activation promotes IL-1β and IL-18 release, gasdermin D cleavage, and pyroptotic cell death, leading to sustained non-infectious inflammation and adverse ventricular remodeling [32].

Unlike atherosclerosis, arteriosclerosis often does not produce stenosis disease which can exacerbate conditions (e.g., hypertension, heart failure) due to reduced vascular compliance [23,24]. Shared features include their strong association with aging and classical cardiovascular risk factors (e.g., diabetes, dyslipidemia, chronic inflammation) and their substantial contribution to cardiovascular morbidity and mortality [24]. When advanced, both processes can lead to overlapping clinical consequences (e.g., heart failure, organ dysfunction) despite arising from partially distinct structural changes in the arterial wall [23,24]. Although IL-1β inhibitors have demonstrated anti-inflammatory benefits in clinical trials, as also discussed in recent reviews, these agents remain investigational and are not routinely used in everyday management of coronary artery disease.

In contrast, arteriosclerosis remains more challenging to treat directly and is generally addressed through blood pressure control, glycemic optimization, and lifestyle interventions, which can attenuate progression of arterial stiffening and reduce global cardiovascular risk [24,33]. Taken together, these mechanisms place atherosclerosis at the core of the cardiovascular disease spectrum, serving as a fundamental driver for multiple downstream clinical manifestations such as coronary, peripheral, and cerebrovascular disease.

#### 3.1.2. Coronary Artery Disease

Coronary artery disease (CAD) is a multifactorial cardiovascular disorder and a leading cause of morbidity and mortality worldwide. From a clinical standpoint, the disease spectrum comprises acute presentations (acute coronary syndromes, ACS) as well as chronic manifestations (chronic coronary syndromes, CCS) [34], and is the third-most common and frequent cause of cardiovascular death. In 2005, it was responsible for approximately 6 million deaths globally, underscoring its substantial clinical and societal burden [35,36]. Also, data from the American Heart Association (AHA) indicates a higher risk of developing coronary heart disease among older men (31%) compared to older women (25.4%) [37]. CAD caused the deaths of 371,506 people in 2022, affecting approximately 1 in 20 adults over the age of 20 (approximately 5%), and in 2023 approximately 1 in 6 deaths from cardiovascular disease occurred among adults under the age of 65 [38].

Most acute coronary syndromes do not result from gradually enlarging, flow-limiting plaques. Instead, they typically arise from disruption of non-obstructive, lipid-rich plaques with thin fibrous caps and a high inflammatory cell burden, leading to thrombosis. Additional contributors include neurohormonal activation (notably the renin–angiotensin–aldosterone system), heightened proinflammatory cytokine signaling, increased production of prostaglandins and thromboxane, and arterial wall remodeling that promotes vessel stiffening, together amplifying susceptibility to endothelial injury, inflammation, and thrombotic events [37,39].

Numerous clinical and epidemiologic studies have shown that the symptomatology of CAD can vary significantly depending on gender, age, and other individual factors. The most common signs reported by patients are chest pain, dyspnea, fatigue, dizziness, nausea, and bloating [40]. However, as highlighted by Lowenstern et al., typical symptoms such as chest pain or dyspnea do not always correlate with the angiographic severity of coronary disease, emphasizing the need for comprehensive clinical assessment and objective testing in suspected CAD [41]. These pathophysiological processes (e.g., neurohormonal activation, proinflammatory cytokine signaling, increased prostaglandin and thromboxane synthesis, oxidative stress, arterial wall structural remodeling) collectively contribute to endothelial dysfunction, impaired vasomotor regulation, and heightened vascular inflammation. The combination of elastin loss, collagen accumulation, RAAS overactivation, and oxidative stress accelerates adverse vascular remodeling, promotes plaque instability, and increases susceptibility to thrombotic coronary events [42,43].

Recent multi-omics studies have refined the molecular understanding of coronary artery disease by integrating genomic, transcriptomic, epigenomic, proteomic and metabolomic data. Leon-Mimila et al. showed that combining genome-wide association study (GWAS) with expression and epigenetic quantitative trait locus analyses, as well as proteomics, can link genetic variants to key processes such as endothelial dysfunction, lipid metabolism, inflammation and extracellular matrix remodeling across cardiovascular phenotypes [44]. Also, Fang et al. used a summary-data-based Mendelian randomization approach to integrate methylation, expression and protein data with large-scale CAD GWAS, identifying genes and pathways consistently supported by colocalization, single-cell RNA sequencing of human coronary arteries and validation in an atherosclerotic mouse model [45]. Together, these multi-omics approaches move beyond simple locus discovery to identify causal networks and actionable molecular targets in CAD, illustrating the potential of systems-level integration for precision cardiovascular medicine.

#### 3.1.3. Peripheral Artery Disease

Peripheral artery disease illustrates how systemic atherosclerosis manifests across different vascular territories, reinforcing its role within a unified cardiovascular spectrum. Peripheral artery disease (PAD) is a common manifestation of systemic atherosclerosis characterized by progressive narrowing or occlusion of the peripheral arteries, predominantly affecting the lower extremities [46,47,48,49].

PAD represents a significant global health burden. An estimated 200 million individuals are affected worldwide, with over 8.5–12 million cases reported in the United States alone [46,50]. The prevalence of PAD increases markedly with age, affecting approximately 15–20% of individuals over 70 years. According to the Global Burden of Disease study, in 2019 there were over 56 million PAD cases among adults aged 40–70 years, and 57 million cases in those over 70 [51]. Although the burden of PAD has increased and preventative treatment measures have varied significantly, the overall rate of major amputations in the United States has declined [50].

PAD shares its major risk factors with other atherosclerotic diseases, including diabetes mellitus, hypertension, hypercholesterolemia, systemic inflammation, and, more recently, clonal hematopoietic syndrome, which are all associated with increased risk of disease onset and progression [46,48,49]. Emerging molecular studies have implicated microRNAs (miRNAs) in the pathophysiology of PAD, such as dysregulation of specific miRNAs (e.g., miR-21, miR-143/145, miR-503) which has been linked to endothelial dysfunction, vascular smooth muscle cell proliferation, impaired angiogenesis, and neointimal hyperplasia, all of which contribute to the development and progression of PAD [48].

These findings not only enhance understanding of disease mechanisms but also suggest novel diagnostic and therapeutic targets. Socioeconomic determinants (e.g., income level, educational attainment, insurance status, limited healthcare access) play a critical role in disease management and outcomes. Patients with lower socioeconomic status are more susceptible to experiencing delayed diagnosis, receiving inadequate preventative care, and having amputations without any previous attempts at revascularization [50,51].

#### 3.1.4. Silent Ischemia

Silent myocardial ischemia (SMI) is a hard-to-detect condition with a prevalence of 2–4% in the general population, with higher rates in patient groups such as those with diabetes or stable angina. It is associated with an increased risk of sudden death and other cardiovascular complications, and studies show that patients with diabetes or cardiac autonomic neuropathy are at higher risk of developing SMI [52].

SMI reflects an imbalance between myocardial oxygen supply and demand that occurs without typical anginal symptoms. Its pathophysiology is multifactorial, involving impaired afferent pain signaling (especially in diabetes and autonomic neuropathy), microvascular dysfunction, transient supply–demand mismatch, and subclinical plaque instability [52,53]. Altered pain perception and central processing, brief and self-limited ischemic episodes, and circadian variations in heart rate, blood pressure, and neurohormonal activation further contribute to the silent presentation of ischemia, with episodes more frequently observed in the early morning hours [53].

A summary of selected key clinical studies illustrating distinct clinical use-cases (diagnosis, prevention, therapy, monitoring, functional outcomes) in atherosclerotic and ischemic cardiovascular diseases is presented in Table 1.

### 3.2. Thromboembolic Disorders

Deep vein thrombosis (DVT) and pulmonary embolism (PE) are the two primary clinical manifestations of venous thromboembolism (VTE). DVT refers to the formation of a blood clot in the deep veins, typically of the lower limbs, while PE occurs when part of this clot dislodges, travels through the bloodstream, and lodges in the pulmonary arteries, obstructing blood flow in the lungs [71,72,73]. Although often considered components of the same disease process, DVT and PE differ in clinical presentation, risk profile, and short-term prognosis [71]. VTE is a frequent vascular condition, with an estimated annual incidence of 1–2 per 1000 individuals in the general population [74]. The incidence of VTE rises with age and is slightly higher in men than women [71,72,75]. DVT is often provoked by transient factors such as surgery, trauma, or immobility, whereas PE may also arise in the context of chronic conditions like heart failure or malignancy [71,72]. A wide spectrum of risk factors contributes to VTE, including acute triggers (e.g., surgery, trauma, hospitalization, acute infection), subacute or intermediate triggers (e.g., systemic inflammation, hormone therapy, pregnancy), and chronic conditions such as obesity, cancer, heart failure, and chronic inflammatory or autoimmune diseases [73,75].

Despite their differences, DVT and PE share many similarities. They are driven by the same underlying pathophysiological mechanisms (e.g., Virchow’s triad of venous stasis, endothelial injury, hypercoagulability) and are treated with similar anticoagulant strategies [71,72,73]. The long-term complications of post-thrombotic syndrome after DVT and chronic thromboembolic pulmonary hypertension after PE are significant, and both conditions are at risk of recurrence if anticoagulation is stopped prematurely [71,72,74]. Although traditionally categorized separately, venous and arterial thrombotic events have the same inflammatory and coagulation pathways, which enable them to be included in an interconnected cardiovascular disease spectrum.

### 3.3. Structural and Valvular Diseases

#### 3.3.1. Valvular Heart Disease

The degenerative, inflammatory, and hemodynamic mechanisms involved in valvular heart disease (VHD) further illustrate how structural cardiac disorders intersect with the broader cardiovascular spectrum. The term VHD refers to the damage caused to one or more cardiac valves (e.g., aortic, mitral, tricuspid, pulmonary), which can result in stenosis or regurgitation, and eventually lead to chamber remodeling, heart failure, arrhythmias, and thromboembolic complications [76].

The burden of VHD increases steeply with age, degenerative aortic stenosis and primary mitral regurgitation are common in high-income countries, but rheumatic valve disease is still the primary cause in many low- and middle-income regions. Epidemiologic data suggests that clinically relevant VHD affects millions of individuals worldwide, yet the actual prevalence may be overestimated due to limited access to echocardiography and under-coding in administrative datasets [77,78].

Symptoms range from exertional dyspnea, angina, syncope, and palpitations to peripheral edema or signs of endocarditis, but many patients remain asymptomatic until advanced disease [79]. Diagnosis relies on multimodal imaging, with transthoracic and transesophageal echocardiography as first-line tools, complemented by CT and cardiac MRI for detailed anatomical and functional assessment when needed [80,81]. This structural disease cluster exemplifies how chronic hemodynamic overload and myocardial–valvular interaction contributes to the global cardiovascular disease continuum.

#### 3.3.2. Rheumatic Heart Disease

Rheumatic heart disease (RHD) exemplifies the contribution of immune dysregulation to the cardiovascular spectrum, linking infection, inflammation, and long-term structural damage. It is an autoimmune disorder that occurs as a complication of *Streptococcus pyogenes* or group A streptococcus infection [82]. RHD is responsible for 1.6% of all cardiovascular deaths, with 306,000 deaths reported annually, mostly among people in low- and middle-income areas [83]. The 2015 Global Burden of Disease Study estimated that there were between 38 and 41 million cases of RHD globally in 2017, with the highest prevalence and mortality rates in Oceania, South Asia, and sub-Saharan Africa [84].

Clinically, RHD is often preceded by acute rheumatic fever, characterized by carditis, polyarthritis, and, in some cases, neurologic or cutaneous manifestations [85,86,87]. If patients suffer from myocarditis, an electrocardiogram (EKG) should be performed to identify possible conduction disturbances and the type of heart block present (grade I, II, or III). A chest X-ray to detect early signs of congestive heart failure or transthoracic echocardiography to detect rheumatic heart disease is also recommended [85]. The most prevalent and deadly complications of RHD include heart failure, pulmonary hypertension, and infective endocarditis. Because of the silent nature of the condition, most patients are diagnosed with the disease and its associated complications at a late stage [85].

Prevention and early detection are therefore central: primary prevention relies on timely antibiotic treatment of streptococcal pharyngitis, while secondary prevention requires long-term antibiotic prophylaxis in individuals with prior rheumatic fever to reduce recurrences and further valve damage [85,86,87]. Advanced valvular disease often necessitates percutaneous or surgical intervention, but access to specialized care is still limited in many high-burden settings [82,83,84].

#### 3.3.3. Congenital Heart Defects

Congenital heart defects (CHD) have a major impact on public health. They are estimated to occur at a frequency of about 8 cases per 1000 live births (about 1%), and about 3 in 1000 newborns require specialized cardiology intervention [88]. Despite improvements in diagnosis and treatment, survival has remained low compared to the general population, especially in complex lesions like truncus arteriosus or single-ventricle physiology. As a result, the number of adults living with CHD has steadily increased; By 2000, approximately one million adults were estimated to have CHD in the United States, and approximately 50% of them had moderate or severe forms [89]. The clinical presentation varies greatly depending on the lesions type and severity, with cases ranging from asymptomatic murmurs to cyanosis, heart failure, growth impairment, and recurrent respiratory infections [90].

The diagnosis of congenital heart disease in both children and adults relies on multimodal cardiovascular assessment. The diagnostic approach relies on multimodal cardiovascular assessment: fetal and postnatal echocardiography are central for anatomical and functional characterization, while ECG, chest radiography, pulse oximetry, and, in selected cases, cardiac catheterization or advanced imaging provide complementary information [89,90]. CHD thus exemplifies how genetic, developmental, and environmental factors converge early in life to shape long-term cardiovascular risk and illustrates the need for lifelong, multidisciplinary follow-up within the cardiovascular disease spectrum [88,89,90].

Table 2 provides a summary of clinical and imaging observations, emphasizing diagnostic challenges, high-risk scenarios, and the need for multimodality imaging and multidisciplinary care.

### 3.4. Inflammatory and Infectious Diseases

The inflammatory and infectious aspects of the cardiovascular spectrum are depicted by endocarditis and pericarditis, which demonstrate how systemic immune activation intersects with structural and hemodynamic pathology [104,105]. Certain forms of both conditions can lead to life-threatening situations and require prompt recognition and customized management. *Staphylococcus aureus* is now the leading causative agent, particularly in healthcare-associated and intravenous drug use (IVDU)-related cases, overtaking the previously dominant streptococcal species [104,106].

In contrast, acute pericarditis is more commonly idiopathic or viral and usually follows a benign, self-limited course, although complications such as cardiac tamponade, recurrent pericarditis, and chronic constrictive forms can occur, especially in secondary causes linked to malignancy, autoimmune disease, or tuberculosis [105,107].

The pathogenesis of IE involves microbial seeding of a damaged or prosthetic endocardial surface, typically following transient bacteremia. The pathogens adhere to fibrin-platelet thrombi, forming vegetations that can embolize and cause systemic complications. *S. aureus*, in particular, possesses virulence factors like fibronectin-binding proteins that facilitate this process [108]. In pericarditis, inflammation may result from direct infection, autoimmune processes, neoplasms, trauma, or post-cardiac injury syndromes. It is typically managed with NSAIDs and colchicine, with corticosteroids or interleukin-1 inhibitors reserved for recurrent or refractory cases. Tuberculous or bacterial pericarditis demands targeted antimicrobial treatment [109]. Notably, both conditions have seen evolving trends in recent years. COVID-19 infection and vaccination have been implicated in triggering pericarditis, particularly in younger males, although outcomes are usually favorable [107]. Meanwhile, IE cases associated with healthcare interventions and prosthetic materials are rising, requiring more vigilant infection control and diagnostic protocols [104,108].

Myocarditis is an inflammatory disease of the myocardium caused by infectious or non-infectious triggers and represents the myocardial counterpart of pericardial and endocardial inflammation within the cardiovascular spectrum [110,111,112]. It is most frequently associated with viral infections (e.g., enteroviruses, adenoviruses, SARS-CoV-2), but can also occur in the context of systemic autoimmune diseases, hypersensitivity reactions to drugs or vaccines, exposure to cardiotoxic toxins, and modern cancer therapies [111,112,113]. The incidence is difficult to determine because many cases remain subclinical, but registry and administrative data suggest that myocarditis is more common in younger individuals, and it is a recognized cause of sudden cardiac death and a precursor of dilated cardiomyopathy [110,111,113]. Initial evaluation typically includes ECG, cardiac biomarkers, inflammatory markers, and transthoracic echocardiography, mainly to exclude alternative structural causes. Cardiac magnetic resonance imaging is the reference non-invasive modality to detect myocardial edema, hyperemia and fibrosis, and to support the diagnosis [110,111,112]. Endomyocardial biopsy remains the histological gold standard and is reserved for selected patients with fulminant presentations, unexplained cardiogenic shock, malignant arrhythmias or suspected specific etiologies in which histology, immunohistochemistry and genetic testing may alter management [111,112,114].

Management of myocarditis is largely supportive and tailored to the clinical phenotype, focusing on guideline-directed therapy for acute and chronic heart failure, treatment of arrhythmias and conduction disturbances, and hemodynamic support in fulminant cases, including temporary mechanical circulatory support when needed [110,112,113]. Prognosis is generally favorable in patients with uncomplicated presentations and preserved left ventricular function, but a substantial minority progress to chronic inflammatory cardiomyopathy, with persistent systolic dysfunction and increased risk of adverse events [111,112,113].

In conclusion, infective endocarditis, pericarditis, and myocarditis are distinct but occasionally overlapping inflammatory cardiac diseases with unique epidemiology, pathogenesis, and management. Timely recognition, accurate diagnosis, and etiology-specific treatment are essential to reduce morbidity, mortality, and long-term complications in both disorders.

### 3.5. Aortic Disorders (Aortic Aneurysm and Dissection)

Aortic aneurysms and dissections (AADs) are life-threatening manifestations of advanced vascular remodeling and structural failure of the aortic wall. An aortic aneurysm is defined as a pathological dilatation of the aorta due to progressive weakening of the wall, whereas aortic dissection occurs when an intimal tear allows blood to enter the media and create a false lumen [115,116,117,118]. Incidence rises steeply with age and is higher in men and in patients with heritable connective tissue disorders such as Marfan, Loeys–Dietz or Ehlers–Danlos syndromes [119,120].

From a pathophysiological perspective, both aneurysms and dissections are driven by medial degeneration, marked by loss of vascular smooth muscle cells (vSMCs), fragmentation of elastin fibers, and inflammatory remodeling of the extracellular matrix [118,121]. vSMCs play a key structural role in the aortic wall, and their dysfunction through apoptosis, phenotypic switching, or contractile failure and weakens aortic integrity [118,122]. Additionally, molecular factors such as programmed cell death (e.g., apoptosis, necroptosis, ferroptosis), cytokine signaling (IL-6, TNF-α), and genetic mutations (e.g., *FBN1*, *ACTA2*, *PLCE1*) have been implicated in disease progression [117,119].

Aortic dissection is often characterized by sudden, severe chest or back pain, which can be described as tearing or ripping, and can also lead to hypotension, syncope, neurological deficits, or signs of organ malperfusion [120]. Aneurysms are often unnoticed until they rupture, which can lead to hypotension, shock, or death [120,123]. Diagnosis relies heavily on imaging, CT angiography is the gold standard, with echocardiography and MRI also used depending on clinical setting and stability. Treatment strategies differ by condition and severity. Acute Type A dissection requires emergency surgery, while Type B dissections are usually managed medically unless complications arise, in which case endovascular repair is favored [116]. Aneurysm repair (either open or endovascular) is recommended when size thresholds or rapid growth are detected. Unfortunately, no current pharmacotherapy has proven effective at halting aneurysm progression, though experimental targets include vSMC survival pathways and inflammatory modulators [118,122]. The link between mental health disorders and substance abuse, particularly stimulants like cocaine, to increased risk and poorer outcomes in aortic dissection is being highlighted by emerging evidence, requiring comprehensive patient management strategies [124].

In summary (Table 3), aortic aneurysm and dissection are linked yet distinct aortic pathologies that share common pathomechanisms involving cellular degeneration, mechanical stress, and genetic predisposition. Also, aortic aneurysms and dissections reflect shared pathways of vascular remodeling, genetic predisposition, and inflammation, connecting them to the systemic continuum of cardiovascular disease.

### 3.6. Rare Cardiovascular Disorders

#### 3.6.1. Cardiac Amyloidosis

Cardiac amyloidosis is a prototypical infiltrative cardiomyopathy in which misfolded proteins aggregate as insoluble fibrils in the extracellular matrix of the myocardium, leading to increased ventricular stiffness, diastolic dysfunction and progressive heart failure [129]. The main types with cardiac involvement are light-chain (AL) amyloidosis and transthyretin (ATTR) amyloidosis, the latter occurring in both hereditary (ATTRv) and wild-type (ATTRwt) forms [129,130,131]. Congestive heart failure, often with preserved ejection fraction, can occur because of cardiac amyloidosis, and electrical conduction disturbances (e.g., atrioventricular blocks) and arrhythmias, particularly atrial fibrillation, can happen, with an increased risk of thromboembolic events [132,133].

#### 3.6.2. Takotsubo Cardiomyopathy (Broken Heart Syndrome)

Takotsubo cardiomyopathy (TC), also known as Takotsubo syndrome (TS), is a transient and reversible systolic dysfunction of the left ventricle that clinically mimics acute myocardial infarction in the absence of obstructive coronary artery disease. The term “Takotsubo” originates from a traditional Japanese octopus’ trap with a round base and narrow neck, resembling the characteristic ventricular shape observed in this condition [134]. Before being formally defined in 1990, various terms were used, but TS has since gained worldwide recognition. Although it can affect individuals of all ethnic groups, the syndrome is less frequent in Hispanics and African Americans. Its prevalence is estimated at approximately 2% of acute coronary syndrome cases, but may reach up to 10% among women, particularly postmenopausal, with a mean age of onset between 65 and 70 years. The incidence of diagnosis increased markedly from 2006 to 2012, largely due to heightened awareness and the widespread use of early coronary angiography. Recurrence occurs in 0–22% of cases, being more common in patients with pheochromocytoma or severe medical conditions [135].

The underlying pathophysiological mechanisms remain incompletely understood. Proposed hypotheses include catecholamine excess, microvascular dysfunction, inflammation, coronary spasm, and estrogen deficiency. The catecholamine hypothesis is the most widely accepted, supported by elevated plasma levels of adrenaline, noradrenaline, and dopamine, which may induce vasospasm and myocardial injury. In postmenopausal women, estrogen deficiency likely amplifies vasoconstrictive stress responses. Imaging studies further suggest roles for inflammation and microvascular impairment. Interestingly, patients with TC exhibit a lower prevalence of diabetes, possibly reflecting an altered autonomic response, an observation referred to as the “diabetes paradox” [136].

Clinically, most patients present with acute chest pain resembling myocardial infarction, although symptoms of heart failure (dyspnea, syncope, pulmonary edema) are also common. A minority remain asymptomatic, with diagnosis established incidentally through routine electrocardiography. Physical findings are often nonspecific, but diaphoresis, hypotension, or signs of acute heart failure may occur. In rare cases, complications such as mitral regurgitation or cardiogenic shock can develop [137].

Diagnosis should be considered in patients presenting with acute coronary syndrome symptoms when clinical and echocardiographic findings are atypical. It is supported by the Mayo Clinic diagnostic criteria, characterized by transient left ventricular dysfunction not explained by coronary obstruction. Notably, TS may coexist with coronary artery disease in up to 15% of cases. Coronary angiography is essential to exclude obstructive lesions, while echocardiography and ventriculography reveal transient wall motion abnormalities. Cardiac magnetic resonance imaging can further differentiate myocardial infarction from reversible myocardial edema by the absence of late gadolinium enhancement [138]. Potential complications include ventricular arrhythmias, left ventricular outflow tract obstruction, atrial fibrillation (paroxysmal or persistent), thromboembolic events, hypotension, and acute heart failure. Recurrence may occur weeks to years after the initial episode [136].

#### 3.6.3. Cardiac Sarcoidosis

Cardiac sarcoidosis (CS) is an inflammatory, infiltrative cardiomyopathy characterized by non-necrotizing granulomatous inflammation that may occur as part of systemic sarcoidosis or, less commonly, as an isolated cardiac phenotype [139,140]. Within the spectrum framework, CS exemplifies how immune-driven myocardial injury can intersect with electrical instability and progressive remodeling, creating overlap with arrhythmic and heart failure domains despite its rare classification [140,141]. Although clinically manifest cardiac involvement is diagnosed in only a minority of patients with systemic sarcoidosis, advanced imaging and autopsy data suggest that clinically silent myocardial involvement is substantially more frequent, highlighting a persistent gap between biological disease burden and routine clinical recognition [140]. The most common clinical expressions of CS include conduction abnormalities, ventricular arrhythmias and sudden cardiac death, and heart failure syndromes, reflecting patchy inflammation and scar formation in the myocardium [140,142].

Diagnosis is challenging because myocardial involvement is focal and dynamic and there is no single definitive noninvasive test. Endomyocardial biopsy is highly specific but has limited sensitivity due to the patchy distribution of granulomas, with improved yield when guided by voltage mapping or advanced imaging [143]. Advanced imaging is central for diagnosis and for phenotyping along an inflammation–fibrosis continuum [141,143]. Cardiac magnetic resonance identifies myocardial injury and replacement fibrosis via late gadolinium enhancement (LGE), typically in patchy, non-ischemic patterns, and LGE carries prognostic information [142,143].

From a precision cardiology perspective, CS is well-suited to mechanism-informed phenotyping because measurable features map directly onto the inflammation–fibrosis axis. Multi-omics profiling may refine immune–inflammatory endotypes and identify pathways linked to adverse remodeling, while AI-enabled integration of ECG and multimodal imaging can support earlier detection, triage, and monitoring as clinician-facing decision support rather than standalone automation [141,142,143].

### 3.7. Major Acute Cardiovascular Events

#### 3.7.1. Cerebrovascular Diseases (Stroke Ischemic/Hemorrhagic, TIA)

Cerebrovascular diseases (CeVDs) refer to a group of disorders that affect blood flow in the brain, typically resulting from either blockage (ischemia) or rupture (hemorrhage) of cerebral vessels. The major clinical presentations include ischemic stroke, intracerebral hemorrhage (ICH), subarachnoid hemorrhage (SAH), cerebral small vessel disease (CSVD), cerebral aneurysms, vascular malformations, and cerebral venous thrombosis [144,145]. These conditions are a leading cause of death and long-term disability globally, especially among aging populations [146,147]. Epidemiologically, ischemic stroke accounts for approximately 80% of all strokes, followed by ICH and SAH [144]. According to the Global Burden of Disease Study, the lifetime risk of stroke is about 25% from age 25 onward [148]. CeVDs are associated with significant health, social, and economic burdens, particularly in low- and middle-income countries where access to acute stroke care and rehabilitation may be limited [144]. The risk factors for CeVDs are both modifiable and non-modifiable. Major modifiable factors include hypertension, diabetes, hyperlipidemia, obesity, smoking, alcohol use, and unhealthy diet, which are often associated with chronic inflammation and oxidative stress [149]. Non-modifiable factors include age, sex, and genetic predispositions, with increasing evidence implicating polygenic and monogenic disorders, such as cerebral autosomal dominant arteriopathy with subcortical infarcts and leukoencephalopathy (CADASIL) and cerebral amyloid angiopathy in specific CeVD phenotypes [145]. Pathophysiologically, CeVDs result from impaired cerebral blood flow, leading to hypoxia, neuronal energy failure, and cell death via mechanisms such as apoptosis, necrosis, ferroptosis, and oxidative stress [149,150]. Endothelial dysfunction, blood–brain barrier breakdown, neuroinflammation, and oxidative injury are central to both acute damage and chronic neurodegeneration. The brain’s vascular system, including its capillary pericytes and glymphatic drainage, is essential for nutrient delivery and waste clearance, and its failure contributes to both vascular and neurodegenerative pathology [151].

Clinical manifestations depend on the vascular territory and pathology. Ischemic stroke may present with sudden focal neurological deficits, whereas hemorrhagic strokes often involve acute headache, vomiting, and altered consciousness. Some cerebrovascular conditions, such as subarachnoid hemorrhage or venous thrombosis, may initially present with thunderclap headache or seizures [148]. Additionally, vascular cognitive impairment is a chronic consequence of recurrent or diffuse cerebrovascular injury [145]. Imaging plays a key role in diagnosis and management. CT and MRI remain essential in acute settings to differentiate ischemic from hemorrhagic stroke. Advanced techniques like diffusion-weighted imaging (DWI), perfusion-weighted imaging (PWI), CT-angiography (CTA), and MR-angiography (MRA) provide detailed insights into cerebral perfusion and vascular anatomy, guiding treatment decisions [146]. Emerging research highlights novel biomarkers and therapeutic targets. Gut microbiota dysbiosis has been implicated in modulating inflammation and immune responses relevant to stroke and ICH, offering new prevention and treatment strategies such as probiotics, fecal transplantation, and dietary interventions [144]. Exosomes and their microRNA cargo are also being studied for their role in intercellular communication and as potential diagnostic and therapeutic tools [152]. Furthermore, PKM2, a glycolytic enzyme, is now recognized as a key regulator of energy metabolism, oxidative stress, and inflammatory pathways in cerebrovascular conditions [150]. Due to their shared developmental and vascular anatomy with the brain, retinal vascular signs, such as arteriolar narrowing and microhemorrhages, are being increasingly used as surrogate indicators of underdiagnosed cerebrovascular disease [147].

In summary, cerebrovascular diseases represent a complex group of disorders with diverse etiologies and clinical outcomes. Cerebrovascular diseases represent a downstream manifestation of systemic vascular dysfunction, aligning seamlessly with the integrated concept of the cardiovascular spectrum. Advances in imaging, molecular biology, and computational tools are improving our understanding of their pathogenesis and paving the way for more personalized and preventive approaches in stroke care.

#### 3.7.2. Sudden Cardiac Arrest

Sudden cardiac arrest (SCA) is the abrupt cessation of cardiac activity, leading to loss of consciousness and absence of circulation. If not treated immediately, it typically results in sudden cardiac death (SCD), which accounts for approximately 15–20% of all deaths in developed countries [153,154]. While often used interchangeably, SCA refers to the event, and SCD to the fatal outcome. These episodes can occur in individuals with or without previously diagnosed heart disease and frequently arise without warning [155].

Epidemiologically, the incidence of SCA varies by population and setting. Globally, out-of-hospital cardiac arrest (OHCA) rates range from 52.5 to 111.9 per 100,000 person-years depending on the region, with the highest incidence in North America and Australia [153]. The etiology of SCA differs with age. In individuals over 35, the predominant cause is coronary artery disease (CAD), responsible for up to 80% of cases [154,156]. In younger people, especially athletes, inherited cardiomyopathies (e.g., hypertrophic cardiomyopathy) and channelopathies (e.g., long QT syndrome, Brugada syndrome) are more common [157,158]. Among athletes, the risk of fatal arrhythmias is increased during vigorous exertion, often unmasking previously silent cardiovascular abnormalities. Notably, basketball, football, and soccer are the sport’s most frequently associated with SCA in young athletes [158].

Clinically, SCA is characterized by sudden collapse, lack of pulse, apnea, and unresponsiveness. If untreated, irreversible brain damage and death occur within minutes. The most common arrhythmias involved are ventricular fibrillation (VF) and pulseless ventricular tachycardia (VT), although non-shockable rhythms such as pulseless electrical activity (PEA) and asystole are increasingly observed, particularly in older or chronically ill patients [155,156]. Immediate CPR and defibrillation are critical for survival. However, bystander response remains inadequate, especially in athletic settings, underscoring the need for widespread AED availability and emergency action plans [158]. Prevention and risk stratification remain central challenges. While reduced left ventricular ejection fraction (LVEF) has traditionally guided decisions for implantable cardioverter–defibrillator (ICD) placement, it is now recognized as an imperfect predictor—particularly in women and younger individuals [156]. Ongoing efforts seek to improve predictive models using imaging, genetic screening, and electrophysiological assessments [154].

Importantly, the impact of SCA extends beyond resuscitation. Survivors frequently experience long-term physical, cognitive, and psychological sequelae, including depression, PTSD, anxiety, and impaired quality of life [159,160]. These effects may persist for months to years’ post-discharge. Unfortunately, structured rehabilitation and follow-up care addressing these multidimensional needs are often lacking. Survivors and families report feeling unprepared for the aftermath of the event, citing a lack of emotional support and continuity in care [160].

In summary, sudden cardiac arrest remains a major global health issue, particularly due to its unpredictable nature, high mortality, and broad impact on survivors and their families. Although early recognition and resuscitation improve outcomes, comprehensive approaches including risk stratification, psychosocial support, and system-wide preparedness, especially in sports and community settings, are crucial to reduce its burden.

#### 3.7.3. Acute Heart Failure and Cardiogenic Shock

Acute heart failure (AHF) is defined as de novo or acutely decompensated heart failure, typically presenting with dyspnea and signs of congestion [161,162,163]. Although the clinical phenotype may look similar, AHF is pathophysiologically heterogeneous because it reflects different underlying cardiac substrates and precipitating factors (commonly including acute coronary syndromes) [161]. Systemic venous congestion is a central feature and results from fluid accumulation and/or redistribution with increased filling pressures and cardio-renal-hepatic interactions that can drive downstream organ dysfunction [162]. Initial in-hospital management is therefore largely syndrome-directed, emphasizing rapid decongestion (e.g., IV diuretics) and supportive measures (e.g., non-invasive ventilation) when respiratory failure is present [162,163]. In hypertensive AHF phenotypes, short-acting vasodilators can be considered to reduce afterload and improve symptoms when blood pressure allows. Before discharge, contemporary pathways emphasize early initiation/optimization of guideline-directed HF therapies and structured early follow-up to reduce post-discharge events [162,163].

Cardiogenic shock represents the most severe end of the acute HF spectrum and is characterized by low cardiac output with sustained tissue hypoperfusion leading to end-organ dysfunction [164,165]. Best-practice approaches emphasize early invasive hemodynamic assessment to define shock phenotype (e.g., LV-, RV- or biventricular-predominant) and guide tailored vasoactive therapy and device selection [166]. When pharmacologic support is insufficient, temporary mechanical circulatory support should be considered in a phenotype-driven manner, ideally within multidisciplinary shock teams and regionalized shock systems of care [165,166].

Within a spectrum-based framework, AHF and CS can be viewed as convergent acute endpoints across multiple etiologic pathways, where multimodal data integration and clinician-facing decision support (including digital/AI workflows) may improve triage and timely escalation.

## 4. Current Emerging Topics in Cardiovascular Research

### 4.1. Precision Medicine and Genomic Insights in Cardiovascular Diseases

Precision medicine in cardiology has evolved from a conceptual framework to a clinically actionable discipline that integrates multiomics and artificial intelligence to guide prevention and therapy [167,168]. This paradigm is echoed by contemporary cardiovascular statements and policy documents, which emphasize that genomic testing, polygenic risk assessment, and AI-enabled decision support are now central to individualized cardiovascular care and population-level prevention strategies [19,169,170,171].

Recent genomic studies demonstrate that cardiovascular diseases are highly polygenic, resulting from the interaction of multiple genetic and environmental determinants [172]. This polygenic architecture is highlighted both in translational reviews and in policy statements that describe a continuum from monogenic to polygenic inheritance and the modifying role of lifestyle and environmental exposures [167,169,170].

Polygenic risk scores (PRS) have emerged as robust tools for quantifying inherited susceptibility by aggregating the effects of millions of common variants associated with coronary artery disease, stroke, and cardiomyopathies [173], and their incorporation into cardiovascular risk prediction models is now a key focus of both scientific reviews and implementation-focused guidance [170,171]. However, conventional PRS models are limited by ancestry bias and feature selection inefficiency. AI-optimized PRS, which integrate clinical, biochemical, and imaging data, significantly improve predictive performance, allowing for early identification of high-risk individuals and personalized prevention strategies [174].

This trajectory aligns with systems and network-medicine approaches that advocate combining large, multi-ancestry genomic datasets with AI and machine learning to enhance cardiovascular risk stratification and to discover new therapeutic targets [19,169]. The integration of genomics with stem cell and functional modeling has deepened mechanistic understanding. Induced pluripotent stem cell platforms now enable patient-specific disease modeling and drug response profiling, allowing genotype–phenotype correlation for arrhythmias, cardiomyopathies, and congenital defects [175].

In parallel, the rise in multiomics, which combine genomic, transcriptomic, epigenomic, proteomic, and metabolomic data, has transformed cardiovascular research. For example, research studies in atherosclerosis show that multiomic integration using machine learning enables accurate molecular phenotyping of atherosclerotic disease, outperforming traditional biomarkers in predicting major cardiovascular events [176]. Aikawa et al. study extend this concept by showing how systems biology and network medicine can integrate multiomic datasets with clinical phenotypes to define disease modules, identify biomarkers, and prioritize drug targets across a spectrum of cardiovascular conditions [19].

At the therapeutic frontier, advances in genome editing (e.g., CRISPR-Cas9) and gene therapy are ushering a new era of “precision cardiology.” Genome editing allows the direct correction of pathogenic mutations in genes such as *PCSK9*, *MYBPC3*, and *LMNA*, offering curative potential for familial hypercholesterolemia and hereditary cardiomyopathies [177,178]. Emerging single-cell genomic technologies now delineate cell-type-specific transcriptional programs within the heart, guiding targeted delivery of gene therapies and uncovering new molecular targets [177]. Furthermore, translational biomarker research links genetic profiles to metabolic and inflammatory pathways, integrating omic data with traditional risk factors to optimize cardiovascular diseases risk prediction and management [12]. Collectively, these advances demonstrate a convergence between genetics, computational modeling, and clinical practice.

### 4.2. Artificial Intelligence and Digital Cardiology

Artificial intelligence and digital health technologies are profoundly transforming cardiovascular medicine, shifting the field from episodic diagnostics to a predictive, data-driven, and patient-centered paradigm. The adoption of machine learning models has been accelerated by the exponential growth of medical data, which has enabled them to extract complex clinically relevant patterns from multimodal sources (e.g., ECG, echocardiography, imaging, wearable sensors) [12,178].

Deep and transformer-based neural networks, such as convolutional neural networks (CNNs) and hybrid CNN-BiLSTM architectures, have demonstrated diagnostic accuracies exceeding 95% in arrhythmia detection, QT interval evaluation, and early identification of left ventricular dysfunction, frequently outperforming expert interpretation while being deployable on low-power wearable devices through edge-end processing and quantization-aware training [179]. Automated chamber quantification, strain analysis, and plaque characterization in echocardiography and cardiac imaging can be facilitated by AI, which improves reproducibility and workflow efficiency while supporting precision risk stratification and prognostic assessment [15].

The development of cardiovascular digital twins, i.e., continuously updated virtual models integrating physiological, imaging, and genomic data, allows for simulation of hemodynamic responses, prediction of decompensation, and optimization of therapeutic strategies, which represents significant progress towards personalized and proactive cardiovascular care [180,181]. Another example is the integration of AI-powered digital stethoscopes (e.g., Eko CORE 500) and portable biosensors with telemedicine systems, which have increased diagnostic accessibility by enabling remote detection of arrhythmias, valvular disease, and heart failure exacerbations, although challenges remain regarding device interoperability, data quality, and standardization [182].

Emerging research in metabolic intelligence, combining physics-based neural networks with real-time metabolic imaging, connects molecular energetics with cardiovascular performance, offering adaptive, physiology-based disease modeling with the potential for dynamic prediction and personalized therapy. Despite technological advances, limitations remain in algorithmic transparency, dataset heterogeneity, and clinical validation due to biases and the “black box” nature of deep models that hinder interpretation and trust [183]. From a regulatory and policy perspective, under the European Medical Devices Regulation, AI-based diagnostic software is classified as a medical device requiring CE certification, clinical validation, and post-market surveillance, while the upcoming Health Technology Assessment Regulation introduces standardized real-world evidence pathways, emphasizing transparency, security, and equality of access [184].

Furthermore, the interdisciplinary convergence of technology and humanism has gained renewed attention as visual and generative art is increasingly used in cardiology to enrich medical education, empathy, and patient recovery, enhancing the human dimension of care in the era of digital transformation [185].

### 4.3. Clinical Implications and Readiness

In practice, we distinguish approaches that are already deployable in routine workflows (e.g., AI-assisted echocardiography, established digital monitoring), those that are emerging with early adoption (e.g., PRS in selected prevention pathways), and those that remain investigational (e.g., multi-omics endotyping, digital-twin models), pending robust prospective validation and governance.

A spectrum-based framework enables practical, cross-phenotype decision support by re-using signals that generalize across traditionally separated diagnoses. First, genomics-driven prevention can leverage polygenic risk scores, optionally integrated with clinical and imaging data, to identify high-risk individuals earlier and intensify preventive strategies before overt disease manifests across vascular territories. Second, AI-enabled triage and early detection using ECG and echocardiography can support rapid in/out workflows and community screening that may precede heart failure or major acute events. Third, multi-omics phenotyping can move beyond single biomarkers by defining mechanistic “treatable traits” that cut across diagnoses, thereby helping prioritize targeted prevention and therapy and enabling longitudinal monitoring of biological response. Across these examples, the spectrum approach emphasizes that multi-omics and AI tools are most useful when embedded as clinician-facing decision support, complementing, rather than replacing, clinical assessment in multimorbid real-world populations.

Practical deployment of spectrum-based multi-omics and AI tools depends on data harmonization and interoperability across sites, including standardized acquisition protocols, consistent labeling, and robust quality control of ECG, imaging, omics, and real-world data streams. Model performance must be demonstrated beyond retrospective benchmarks through external and prospective validation, with ongoing post-deployment monitoring for dataset shift and safety in real-world workflows.

Key barriers include algorithmic transparency and the nature of deep models, which can limit interpretability and clinician trust, as well as dataset heterogeneity and bias that may compromise fairness across diverse populations. Workflow integration is essential to avoid alert fatigue and to ensure these systems function as clinician-facing decision support rather than standalone automation. Finally, implementation must align with governance and regulation, because under the European Medical Devices Regulation, AI-based diagnostic software is regulated as a medical device requiring CE certification, clinical validation, and post-market surveillance, and EU health technology assessment pathways increasingly emphasize standardized real-world evidence, transparency, security, and equitable access.

## 5. Conclusions

The cardiovascular disease landscape is being fundamentally reshaped by advances in multi-omics and artificial intelligence, which enable more precise risk stratification, earlier detection, and more personalized therapeutic strategies. Despite these technological advances, CVD remains one of the leading causes of death and disability worldwide, largely due to delayed diagnosis and suboptimal long-term management of cardiovascular risk factors. Importantly, although multi-omics and artificial intelligence have improved risk stratification and personalized care in CVD, significant clinical challenges persist. These technologies should complement, rather than replace, careful clinical assessment, as thorough history-taking and physical examination remain essential for optimal patient management. Future efforts should focus on integrating multi-omics and artificial intelligence tools into clinically validated workflows, supported by prospective studies, standardized data frameworks, and clinician education, to enable their responsible adoption in everyday cardiovascular practice.

## 6. Future Perspectives

The integration of heterogeneous data across the entire disease spectrum will be crucial for future advancements in cardiovascular medicine. While traditional risk factors and imaging findings remain fundamental in clinical practice, their predictive power is limited when considered alone. Artificial intelligence and machine learning are expected to play an important role in translating this growing complexity into clinically actionable information, supporting rather than replacing clinical decision-making. Artificially enhanced imaging, automated ECG interpretation, and decision-support systems integrating multi-omics and clinical data have the potential to become routinely embedded within clinical workflows to assist clinicians in risk stratification and treatment planning.

Ensuring transparency, robustness, and fairness of these models across diverse represents a major challenge and is essential to prevent the amplification of existing healthcare disparities, while maintaining interpretability and clinical accountability. At the mechanistic level, systems biology and network-based approaches are likely to further refine how cardiovascular diseases are defined and managed, offering clinicians deeper insight into shared disease pathways.

Multi-omics-derived signatures may guide increasingly individualized treatment strategies, including drug repurposing, combination therapies and adaptive treatment algorithms that evolve as new data emerge. Importantly, education of both clinicians and patients will be critical to ensure that novel technologies are appropriately understood, trusted, and applied as decision-support tools rather than perceived as black-box replacements for clinical judgment.

In the end, seeing cardiovascular conditions as a continuum that is linked by shared mechanisms and informed by multilayer data could lead to a transition from reactive, event-driven care to proactive, and precision-based prevention. Successful implementation of such an integrated framework could reduce the global burden of cardiovascular disease and help narrow inequities in outcomes across populations and healthcare systems.

## Figures and Tables

**Table 1:** Evidence summary in atherosclerotic and ischemic cardiovascular diseases, grouped by clinical use-case (diagnosis, prevention, therapy, monitoring, functional outcomes, investigational outcomes trials).

Clinical Disease	Main Findings	Clinical Use-Case	References
Atherosclerosis and arteriosclerosis	In patients over 75 years, moderate-intensity statin plus ezetimibe provides similar cardiovascular protection to high-intensity statin therapy, with fewer intolerance-related discontinuations and less new-onset diabetes, while maintaining LDL-C reduction.	Prevention	[54]
	Long-term *PCSK9* inhibition with Evolocumab safely maintains LDL-C reduction over more than 8 years and reduces cardiovascular death, myocardial infarction, and stroke; earlier initiation yields greater clinical benefit.	Therapy	[55]
	Six months of high-intensity rosuvastatin therapy reduces 18F-NaF PET-CT plaque uptake by ~19%, suggesting decreased plaque metabolic activity and microcalcification stabilization.	Monitoring	[56]
	The SURPASS-CVOT trial compares Tirzepatide versus Dulaglutide for major adverse cardiovascular events in patients with type 2 diabetes and established atherosclerotic cardiovascular disease.	Investigational outcomes trial	[57]
Coronary artery disease	A high-sensitivity cardiac troponin I concentration <5 ng/L identifies a low-risk group among patients with suspected acute coronary syndrome, suitable for early discharge and associated with a very low rate of adverse cardiac events.	Diagnosis	[58]
	High-dose atorvastatin therapy reduces major cardiovascular events compared with standard-dose pravastatin after acute coronary syndromes, supporting the benefit of intensive LDL-C lowering.	Therapy	[59]
	Intensive lifestyle modification (e.g., plant-based diet, exercise, stress management, smoking cessation) over 5 years can induce regression of coronary atherosclerosis in selected patients, even without lipid-lowering drugs.	Prevention	[60]
Peripheral artery disease	High-intensity home-based walking exercise significantly improves 6-min walk distance compared with low intensity exercise or control.	Functional outcomes	[61]
	A behavioral home-based walking intervention did not significantly improve walking distance over usual care; adherence appears critical to effectiveness.		[62]
	EMINENT trial: a paclitaxel-eluting stent (Eluvia) achieves higher 12-month patency than bare-metal stent and provides sustained clinical benefit.	Therapy	[63]
	Rivaroxaban 2.5 mg BID plus aspirin reduces combined MACE and MALE versus aspirin alone in symptomatic PAD, with an acceptable bleeding risk.		[64,65]
	In patients with PAD and type 2 diabetes, liraglutide improves transcutaneous oxygen pressure, C-reactive protein and albuminuria.		[66]
Silent myocardial ischemia	Relatively prevalent in high-risk groups and is associated with increased risk of major cardiovascular events, underscoring the need for systematic detection and risk-factor control. Asymptomatic ischemia is most often detected by resting ECG, stress testing, ambulatory ECG or imaging in high-risk individuals.	Detection	[67,68,69,70]

**Table 2:** Evidence summary in structural and valvular heart diseases.

Clinical Disease	Main Findings	References
Valvular heart disease	Echocardiography, with or without contrast, is essential for evaluating valve morphology and can detect rare lesions such as valvular blood cysts.	[91]
	Ultrasound screening can identify valvular abnormalities even in asymptomatic individuals, including twins, suggesting a possible role of genetic and intrauterine factors in congenital valve defects.	[92]
	Cardiovascular magnetic resonance (CMR) is recommended in patients at risk of heart disease to improve the detection of myocardial fibrosis and structural remodeling associated with valve disease.	[93]
	Infective endocarditis caused by uncommon organisms (e.g., *S. moniliformis*) is diagnostically challenging and requires close clinic–microbiological collaboration.	[94]
	Valvular disease during pregnancy requires careful monitoring; severe mitral stenosis warrants preconception counseling due to the risks related to tachycardia, hypervolemia, and hypercoagulability.	[95]
Rheumatic heart disease	Recurrent acute rheumatic fever may occur in adults despite apparently completed secondary prophylaxis, indicating that extended antibiotic prophylaxis should be considered in selected high-risk patients to prevent recurrences and progressive valve deterioration.	[96]
	Early diagnosis, long-term prophylaxis, and continuous follow-up are crucial to avoid progression of valve disease, especially in disadvantaged communities where RHD burden remains high.	[97]
	Rare coexistence of RHD with systemic lupus erythematosus illustrates the diagnostic challenges in overlapping autoimmune conditions and the need for multidisciplinary assessment and early imaging.	[98]
Congenital heart defects	Severe neonatal CHD that is not amenable to immediate surgical repair requires symptomatic stabilization, careful hemodynamic management, and specialized follow-up.	[99]
	Postpartum hypertension and dyspnea may unmask previously undiagnosed adult CHD; complex lesions such as aortic coarctation with persistent ductus arteriosus can initially be misinterpreted as pulmonary embolism. Persistent postpartum symptoms should prompt evaluation for underlying CHD and early differentiation from pulmonary embolism and other acute conditions.	[100,101]
	Cardiogenic shock in infants can be the first manifestation of complex CHD (e.g., incomplete Shone complex); point-of-care ultrasound may facilitate early recognition and timely intervention.	[102]
	Complex congenital anomalies (e.g., tricuspid atresia with additional defects) require urgent diagnosis, atrial septostomy when indicated, and coordinated surgical planning within a multidisciplinary team to optimize neonatal survival.	[103]

**Table 3:** Evidence summary in aortic disorders.

Clinical Disorder	Main Findings	References
Aortic aneurysm and dissection	■ The use of fluoroquinolone was not associated with an increased risk of aortic aneurysm or dissection in a large Taiwanese cohort, suggesting that current safety warnings may require refinement.	[125]
	■ Painless acute aortic dissection represents a clinically important subset of cases and is associated with higher mortality, underscoring the need for high suspicion even in the absence of chest pain.	[126]
	■ Acute Type A aortic dissection during late pregnancy can be successfully managed with a coordinated multidisciplinary approach combining cesarean section and immediate aortic repair, highlighting pregnancy-related hemodynamic and hormonal triggers.	[127]
	■ A novel single-branched aortic stent graft for Type B dissection showed high technical success, low early mortality, and good 1-year survival, preserving left subclavian artery flow and providing a safe minimally invasive alternative in selected patients.	[128]

## Data Availability

The original contributions presented in the study are included; further inquiries can be directed to the corresponding author.
